# Testing the Association Between Low Back Pain Intensity and Core Muscle Strength in Postpartum Women with Different Delivery Modes: An Analytical Cross-Sectional Study

**DOI:** 10.3390/jcm14186505

**Published:** 2025-09-16

**Authors:** Mohamed G. Ali, Amel M. Yousef, Mohammed A. M. Sarhan, Reem M. Alwhaibi, Hoda M. Zakaria, Abeer A. Mohammed, Walaa M. Ragab, Rehab S. Mamoon, Mohammad Auais

**Affiliations:** 1 Department of Physical Therapy for Women’s Health, Faculty of Physical Therapy, South Valley (Qena) University, Qena 83523, Egypt; 2 Department of Physical Therapy for Women’s Health, Faculty of Physical Therapy, Cairo University, Giza 12613, Egypt; amel.yousef@cu.edu.eg; 3 Department of Physical Therapy for Musculoskeletal Disorders, Faculty of Physical Therapy, Suez Canal University, Ismailia 41522, Egypt; 4 Department of Rehabilitation Sciences, College of Health and Rehabilitation Sciences, Princess Nourah bint Abdulrahman University, Riyadh 11671, Saudi Arabia; 5 Department of Physical Therapy for Neurology, Faculty of Physical Therapy, Cairo University, Giza 12613, Egyptwalaa.ragab@cu.edu.eg (W.M.R.); 6 Department of Physical Therapy, Faculty of Allied Medical Sciences, Isra University, Amman 11622, Jordan; 7 Department of Physical Therapy, College of Medical Rehabilitation Sciences, Taibah University, Medina 42353, Saudi Arabia; 8 Department of Rehabilitation Sciences, College of Health Sciences, Qatar University, Doha 2713, Qatar; 9 Department of Physical Therapy, School of Rehabilitation Therapy, Queen’s Health Sciences, Queen’s University, Kingston, ON K7L 3N6, Canada

**Keywords:** cesarean delivery, core muscle strength, postpartum low back pain, transversus abdominis muscle, vaginal delivery

## Abstract

**Background/Objectives**: Postpartum women frequently experience nonspecific low back pain (NSLBP), yet the impact of delivery mode on the function of local core muscles, particularly the transversus abdominis (TrA) and lumbar multifidus (LM), is not well understood, limiting the development of targeted rehabilitation strategies. To compare NSLBP intensity and TrA and LM strength in women who underwent cesarean delivery (CD) or vaginal delivery (VD), and to examine the associations between pain intensity and muscle strength. **Methods**: An analytical cross-sectional study was conducted on 36 women divided into two groups: 18 who underwent CD (Group A) and 18 who underwent VD (Group B). NSLBP intensity was assessed using the visual analogue scale, while TrA and LM strength were measured via a pressure biofeedback unit. **Results**: The two groups showed non-significant differences in age (*p* = 0.342), BMI (*p* = 0.429), or parity (*p* = 0.894), confirming comparable baseline characteristics. NSLBP intensity was significantly higher in the CD group (*p* = 0.000), and they exhibited weaker TrA (*p* = 0.009) strength than the VD group; however, there was a non-significant difference in LM strength (*p* = 0.602). The Spearman correlation analysis revealed non-significant associations between NSLBP intensity and TrA and LM strength in the CD group (*p* = 0.702, 0.129, respectively) and in the VD group (*p* = 0.149, 0.877, respectively). **Conclusions**: Women undergoing CD experienced higher NSLBP intensities and weaker TrA strength than those undergoing VD, while LM strength remained similar between groups. However, NSLBP intensity showed non-significant associations with TrA or LM strength in either group, suggesting that other biomechanical or neuromuscular factors may contribute to the increased post-CD NSLBP. These findings highlight the need for targeted rehabilitation strategies beyond core muscle strengthening alone.

## 1. Introduction

Cesarean delivery (CD) refers to a mode of childbirth where one or more fetuses are delivered through incisions made in both the abdomen and uterus [1]. There are two abdominal incisions for CD: longitudinal (midline) and lower transverse incisions [2]. The lower transverse incision is the most commonly used for CD [3], allowing access for rectus abdominis separation after the sharp incision through the myofascia of the internal oblique, external oblique, and transversus abdominis muscles [4]. CD can be considered the most frequent major surgical operation performed globally [5]. The CD rate continues to be of global concern because of its constant rise [6]. VD is the natural delivery mode that involves passing the newborn through the normal birth canal (the vagina), generally without the need for extensive medical interventions [7]. However, VD offers several advantages over cesarean delivery (CD), such as lower infection rates, reduced blood loss, shorter hospital stays, lower economic costs, and quicker recovery times [8].

Globally, CD rates have increased from ~7% in 1990 to ~21% in recent estimates and may approach ~28–30% by 2030, with particularly high projections in parts of Asia, Northern Africa, and Latin America; these trends highlight the need to understand postpartum musculoskeletal complications across delivery modes [9]. According to the Central Agency for Public Mobilization and Statistics of Egypt (CAPMAS Egypt), the rate of CD has increased to reach about 72% of all deliveries in 2021, up from 52% in 2014 [10]. This rate is significantly higher than the Canadian CD rate of 28.2% [11]. The high CD rate in Egypt may be partly due to financial incentives, as CD tends to receive higher reimbursement than vaginal delivery (VD) [12]. Other factors contributing to the increased CD rate may include maternal overweight/obesity and obstetricians’ preference for managing their time, as an uncomplicated CD can be completed in a relatively shorter time compared to VD [13].

Nonspecific low back pain (NSLBP) is defined as pain between the lower ribs and the inferior gluteal folds, with or without leg symptoms, in the absence of a specific underlying pathology [14,15]. Early postpartum NSLBP is common and clinically significant, yet its mechanisms remain unclear. Its prevalence in women undergoing CD was 56.67%, which was greater than the 33.33% prevalence in women undergoing VD [16]. The increased vulnerability to postpartum LBP may be attributed to stressful positions during either lactation or delivery, awkward posture, overweight or obesity, pregnancy, and lactation-related osteoporosis, needle trauma from obstetric anesthesia, and a history of pre-pregnancy and pregnancy-related LBP [17,18,19,20]. Despite the lower risk of pelvic floor dysfunctions associated with CD compared to VD [21], CD may contribute to a decrease in abdominal muscle strength, an increased waist-to-fat ratio [22,23], and a decrease in voluntary intra-abdominal pressure [24]. The TrA, one of the abdominal muscles, is prone to weakness following CD due to changes in fascial gliding and muscle thickness [25].

The core muscles consist mostly of four muscles. The lumbar multifidus (LM) and transversus abdominis (TrA) muscles are the two deep local stabilizers, while the pelvic floor muscles and the diaphragm act as synergists [26]. Core muscle strength can be described as the ability of the core to create and maintain forces actively to provide spinal stability through regulating intra-abdominal pressures [27,28]. The spine is unloaded by the slight rise in intra-abdominal pressure caused by TrA activation [29]. Core muscle weakness may be considered a predictor (risk factor) for low back pain (LBP) in athletes [30].

Even though Chang and colleagues, in their 2015 systematic review, confirmed the positive effect of local core muscle strengthening exercises on reducing the severity of chronic LBP [31], previous studies have also examined the association between LBP and either a weak lumbar multifidus or a delayed activation of the transversus abdominis in various musculoskeletal disorders [32,33]. However, despite the rising rates of CD, it remains underexplored whether the mode of delivery is associated with early postpartum NSLBP intensity and local core muscle strength (TrA, LM), thereby limiting the development of targeted rehabilitation pathways. To our knowledge, this is the exclusive study that assessed NSLBP intensity and the strength of the local core muscles (TrA & LM) in women who underwent CD or VD, and examined the association between NSLBP intensity and core muscle strength in postpartum women to fill the gap in this area.

The null hypothesis of this study assumed that women who underwent CD or VD would not show a significant increase in their NSLBP intensity or a significant decrease in their core muscle strength. Additionally, it assumed there would be no association between NSLBP intensity and core muscle strength. Prior findings on postpartum TrA/LM function are heterogeneous across samples, timing, and measurement methods, yielding uncertainty about the expected direction and magnitude of effects at 6–12 weeks postpartum. Accordingly, we prespecified a conservative null hypothesis (no between-group differences; no associations between NSLBP and TrA/LM strength), allowing the data to adjudicate the presence and direction of effects without directional bias.

## 2. Materials and Methods

The study design is an analytical cross-sectional design with retrospective exposure (delivery mode) classification. This study was conducted at the teaching hospitals of South Valley University (SVU), Qena Governorate, Egypt. The study was conducted between 28 June 2021 and 5 August 2023. Data access for research purposes was initially granted between June and October 2021, following the first ethical approval (No.: P.T.REC/012/001954, dated 6 May 2018). Due to administrative delays, the practical phase was temporarily paused and later resumed between May and August 2023. To ensure continued compliance with institutional regulations, the study received renewed approval from the Faculty of Physical Therapy’s International Ethical Committee, Cairo University (No.: P.T.REC/012/005482, dated 10 September 2024). This research proposal was registered on ClinicalTrials.gov with ID: NCT05493891. A documented consent form was gathered after a comprehensive illustration before the beginning of the study.

Personal data, including height, body weight, body mass index (BMI), history of specific low back pain (LBP), and parity (defined as the number of times a woman has given birth to a newborn at 24 weeks of gestation or later, whether alive or stillborn [34]), were collected before participant assessment.

The sample size for this study was determined using the G*Power analysis Version 3.1.9.7. (*t*-test family) based on the primary outcome, the visual analogue scale (VAS) scores of NSLBP. The effect size (Cohen’s d = 1.14) was derived from a prior study by Nawshin and Sanam [35]. In this study, postpartum NSLBP VAS scores were reported for the CD group: Mean ± SD: 5.7 ± 1.7 (*n* = 62), and for the VD group: Mean ± SD: 3.9 ± 1.3 (*n* = 31). Using a statistical power of 0.95, an alpha level of 0.05, and a 1:1 allocation ratio, the required total sample size was calculated to be 36 participants, 18 for each group.

A total of 56 women were recruited for this study; 36 women met our inclusion criteria and completed this study. The subjects were classified into two groups: Group A consisted of 18 women who gave birth via CD, and Group B included 18 women who gave birth via VD without any previous CD or abdominal surgery. Women in Groups A and B were either primiparous or multiparous.

To reduce bias and control for confounders of NSLBP in this study, we exclusively included women aged 18 to 35 years with a body mass index (BMI) ranging from 19.1 to 29.5 kg/m^2^. None of the participants were engaged in physical training programs either during the antenatal or postpartum period. Additionally, we had no conflicts of interest, and a detailed record of each subject was maintained.

The exclusion criteria were: women who were not able to complete assessment processes, women with a BMI higher than 29.5 kg/m^2^ or lower than 19.1 kg/m^2^, women younger than 18 years old or older than 35 years old, women with neuropathic pain or pain radiating to the legs (e.g., radiculopathy) due to specific dysfunctions like lumbar disc prolapse, and women who complained of scoliosis, or spondylolisthesis.

Assessment of women in both groups was performed between 6 and 12 weeks postpartum, with an average of 8 weeks (the index date). The postpartum time was not included as a covariate in the final model, as the relatively narrow window was intended to minimize variability. This assessment period lies beyond the puerperium, which, according to the World Health Organization (WHO), begins immediately after delivery and lasts for approximately 6 weeks (42 days) postpartum [36]. This index date was determined based on the return of many pregnancy-related anatomical and physiological changes to the pre-pregnant state by the end of the 6th week postpartum [37].

### 2.1. Variables of Interest

The independent variables of this study were CD and VD. On the other side, the dependent variables were NSLBP intensity, the primary outcome [measured in a continuous number], and the muscular strength of these core muscles: TrA and LM, secondary outcomes [measured through the determination of maximum voluntary isometric contraction (MVIC) in mmHg for 10 seconds]. MVIC is a highly reliable, standardized, and objective method used to quantify and assess muscle strength [38].

### 2.2. Materials for Evaluation

1. Visual Analogue Scale (VAS): this scale was applied to estimate the severity or intensity score of NSLBP measured by a 10 cm continuous scale for women in the three groups.

The VAS is a highly reliable scale to estimate the intensity of pain, with an intraclass correlation coefficient (ICC = 0.97) [39]. Also, it is a valid scale used for measuring LBP intensity, with Pearson’s correlation coefficient (r = 0.92) when correlated with the Brief Pain Inventory (BPI), and r = 0.75 when correlated with the Modified Oswestry Disability Index (MODI) [40].

After explaining the proper use of VAS as well as its importance in pain measurement to the participants, a score was chosen by making a handwritten mark on the line to describe each subject’s intensity of NSLBP, and the score was recorded in an individualized sheet. The 10 cm scale represents a continuum line between zero, which indicates no pain, and ten, which indicates the worst possible pain [41].

2. Pressure Biofeedback Unit (PBU): It was used to measure the maximal voluntary isometric contraction (MVIC) of TrA and LM muscles for women in the three groups.

The Pressure biofeedback unit (PBU) showed very good to excellent inter-rater and intra-rater reliability for the measurement of TrA activation. The Intraclass Correlation Coefficient (ICC) values for reliability were 0.87 and 0.86, respectively [42]. Also, PBU showed acceptable construct validity for the measurement of TrA activation with Pearson correlation calculations (r) ranging from 0.48 to 0.90 [43,44,45]. PBU is also a reliable tool to measure LM MVIC, with intra-rater reliability ICC agreement of 0.99 and standard error of measurement (SEM) equal to 3.83. Also, when it was correlated to Electromyography, it showed high validity with Pearson correlation calculations (r) of 0.997 [46].

The PBU utilized in this study (Chattanooga Group Inc., Hixson, TN 37343, USA) consisted of a sphygmomanometer gauge, a catheter, and a three-chamber pressure cell filled with air. The PBU’s pressure cell was composed of latex-free rubber, and the cell’s unfolded dimension was 16.7 × 24.0 cm. The gauge has a range of 0 to 200 mmHg and was calibrated to intervals of 2 mmHg. When the subject moves, this produces volume variations in the pressure cells expressed on the sphygmomanometer gauge. The pressure cell was inflated to a pressure of 70 mmHg before data collection, as it was recommended by Ramos et al. [47].

In the case of abdominal drawing-in maneuver (ADIM), supine, prone, or sitting positions can be taken when assessing core muscle strength with the PBU. The subject’s position during this study was the sitting position because it is a fundamental functional position, in addition to the supportive evidence for its accuracy during the assessment of TrA and LM strength when correlated to Electromyography findings [48].

For assessment of TrA strength, the subject was seated upright against the chair’s back, one hand on the gauge, and both feet resting on the floor. The PBU’s pressure cell was fitted to the lumbar spine. To contract TrA, the subject slowly drew her lower abdomen towards the back, as described by Li et al. [48]. To measure TrA MVIC, the subject was encouraged by the researcher and asked to hold TrA contraction for 10 s while maintaining the increase of 2–4 mmHg. The researcher palpated the abdomen just medial to the anterior superior iliac spine to feel TrA activation. The average of three consecutive readings was recorded. More than a 4-mmHg pressure indicates contraction of other abdominal muscles like rectus abdominis and obliques rather than the TrA alone [49].

For assessment of LM strength, the subject was seated as during the TrA assessment; however, the PBU’s pressure cell was fitted between the medial borders of the scapula. To activate the LM, the subject slowly extends her lumbar spine as described by Li et al. [48]. Also, the researcher encouraged the subject to hold LM contraction for 10 s while maintaining the increase of 2–4 mmHg. The researcher palpated the paraspinal muscles to feel the indirect LM activation. Three repeated measurements were taken for each muscle test, and the mean value was used to reduce measurement error.

In the present study, we deliberately focused on the TrA and LM rather than the diaphragm, pelvic floor muscles, and global core stabilizers for several reasons: first, the TrA and LM are the two most consistently studied deep stabilizers in the literature on nonspecific low back pain. Second, we utilized a pressure biofeedback unit, which has demonstrated high reliability and validity for assessing TrA and LM activation in both clinical and research settings. In contrast, accurate assessment of other core muscles, such as the diaphragm and pelvic floor, would require specialized tools (e.g., ultrasound imaging, intravaginal or intrarectal probes, or spirometry-based measures) that were beyond the resources available for this project.

### 2.3. Data Collection

Data were screened for normality assumption using both Kolmogorov–Smirnov and Shapiro–Wilk tests. They indicated only normal distribution (i.e., *p* > 0.05) in these two variables; age in years, and the body mass index (BMI) in kg/m^2^, which allowed us to conduct parametric analysis for them. On the other side, they indicated non-normal distribution (i.e., *p* ≤ 0.05) in the following variables: parity (number of deliveries), visual analogue scale (VAS), the maximum voluntary isometric contraction of transversus abdominis (TrA MVIC), and the maximum voluntary isometric contraction of the lumbar multifidus (LM MVIC), which allowed us to conduct non-parametric analysis for them.

### 2.4. Statistical Analysis

The statistical analysis for the study variables was managed using the statistical SPSS Package program, version 25 for Windows (SPSS, Inc., Chicago, IL, USA). The consecutive statistical processes were managed:

- Quantitative descriptive statistics data: The mean and standard deviation (SD) for the following variables were determined: physical characteristics of subjects (age, BMI, and Parity or number of deliveries), VAS, TrA MVIC, and LM MVIC.
- The Unpaired *t*-test: It was utilized for the mean comparison of the normally distributed variables (age and BMI) between the two groups.
- The Mann–Whitney U test: It was utilized for the mean rank comparison of the non-normally distributed variables (Parity, VAS, TrA MVIC, and LM MVIC) between the two groups.
- Spearman’s Correlation test: This analysis measured the association between NSLBP VAS scores and each core muscle strength in every group.
- Multilinear Regression Analysis: This analysis examined the relationship between Age, BMI, Parity, TrA MVIC, LM MVIC, and VAS scores of NSLBP.
- At the 0.05 level of probability, all statistical analyses were significant (i.e., *p* ≤ 0.05).

## 3. Results

### 3.1. Physical Characteristics of Participants

A total of 36 women completed this study. They were classified into 2 groups: Group A (18 women who underwent CD) and Group B (18 women who underwent VD).

The mean ± SD values of age measured in years for groups A and B were 25.8 ± 5.6 and 27.3 ± 3.5, respectively, with a small effect size of −0.32. The mean ± SD values of BMI measured in kg/m^2^ for groups A and B were 24.9 ± 3.1 and 24.2 ± 2.4, respectively, with a small effect size of 0.25. The mean ± SD values of the number of deliveries (parity) for group A and group B were 2.2 ± 1.2 and 2.2 ± 1.3, respectively, with an effect size of 0 (Table 1).

### 3.2. The Mann–Whitney U Test

#### 3.2.1. The Visual Analogue Scale (VAS)

The mean ± SD values of VAS for the A and B groups were 5.1 ± 1.5 and 3.4 ± 0.8, respectively. The Unpaired *t*-test indicated a significant difference between the 2 groups (*p* = 0.000; *p* ≤ 0.05) (Table 2). The effect size estimation for the VAS scores using Cohen’s d revealed a very large effect size of 1.4. Also, the post hoc power analysis of the VAS scores revealed a power of 98.9%.

#### 3.2.2. Transversus Abdominis (TrA) and Lumbar Multifidus (LM) Maximum Voluntary Isometric Contraction (MVIC)

The mean ± SD values of TrA MVIC for the A and B groups were 2.8 ± 1.1 and 3.7 ± 0.5, respectively. The mean ± SD values of LM MVIC for the A and B groups were 3.7 ± 0.6 and 3.7 ± 0.5, respectively. The Unpaired *t*-test indicated significant differences between the 2 groups in the TrA MVIC and LM MVIC (*p* = 0.009 and *p* = 0.602; *p* ≤ 0.05), respectively (Table 3). The effect size estimation for the TrA MVIC scores using Cohen’s d revealed a very large effect size of 1.1. Also, the post hoc power analysis of the TrA MVIC scores revealed a power of 80.2%. The effect size estimation for the LM MVIC scores using Cohen’s d revealed no effect size of 0.

### 3.3. Spearman’s Correlation Test

Spearman’s correlation test indicated non-significant associations between NSLBP and TrA strength in CD women (rs = 0.097, *p* = 0.702) and between NSLBP and LM strength (rs = −0.371, *p* = 0.129). In VD women, NSLBP was not significantly associated with either TrA (rs = 0.354; *p* = 0.149) or LM strength (rs = −0.039; *p* = 0.877) (Table 4).

### 3.4. Multiple Linear Regression Analysis

The model exhibited an R-value of 0.443, indicating a weak association between the predictors and the dependent variable (VAS scores of NSLBP). The R^2^ value of 0.196 suggests that only 19.6% of the variance in VAS scores can be explained by the independent variables (Age, BMI, Parity, TrA MVIC, and LM MVIC) with an adjusted R^2^ of 0.062, indicating a poor model fit. The ANOVA results revealed that the overall model was not statistically significant (F (5, 30) = 1.464, *p* = 0.231), meaning the predictors collectively do not explain a significant proportion of variance in VAS scores (Table 5).

Among the individual predictors, BMI had the highest standardized beta coefficient (β = 0.353, *p* = 0.087), suggesting a trend toward a positive association with VAS, though it did not reach statistical significance. Other predictors, including Age (*p* = 0.915), Parity (*p* = 0.638), TAMVIC (*p* = 0.205), and LMMVIC (*p* = 0.715), showed no significant contributions to the model (Table 6).

## 4. Discussion

NSLBP is a frequent complaint among postpartum women, often leading them to seek physiotherapy care [50]. This study showed significant increases in NSLBP intensity in women undergoing CD compared to VD. The increased intensity of postpartum NSLBP may result from hormonal changes during pregnancy and a lack of ergonomic consideration when lifting and carrying the baby. Specifically, the significant rise in NSLBP among women who underwent CD could be due to the increased postural stress on the back muscles, caused by compensatory movements to avoid placing strain on the cesarean section incision, or due to changes in the contractile and viscoelastic properties of lumbar paravertebral muscles [51]. This study’s findings agree with the findings of Chia et al. [52], who reported a higher risk of LBP after experiencing CD than VD. Also, our findings run in line with the findings of Rasheed et al. [53], who found the incidence of recurrent LBP (which begins during pregnancy and continues after delivery up to 6 months) was higher following CD when compared to that of VD.

The results of this research indicated a significant decrease in the TrA strength in women who underwent CD when compared to those who underwent VD. The interpretation of this significant decrease in TrA strength may be attributed to surgical trauma to the TrA muscle and its fascia. This trauma likely causes alterations in the fascia, leading to a notable decrease in the muscle’s passive tension. Total muscle tension is derived from both the active tension generated by the muscle tissue and the passive tension produced by its related connective tissue (tendon and fascia) [54,55]. This result agrees with the results of Fan et al. [24], who found thickening of the anterior and posterior fascia of TrA after CD and concluded that after CD, alteration of fascial gliding and abdominal muscles thickness–including TrA–may cause muscular deficits and weakness. On the other side, our results disagree with Kuciel et al. [56], who found that surface Electromyography findings of TrA muscle did not indicate a significant difference between women who underwent CD, and VD.

To the best of our knowledge, this is the first study to assess LM muscle strength following CD and VD. Our results revealed a non-significant decrease in LM MVIC in women who underwent CD compared to those who had VD. This finding can be explained by several factors. First, the LM is less directly affected by abdominal incisions since it is a deep posterior spinal stabilizer, unlike TrA, which is directly disrupted during CD. Second, women who underwent CD may have unconsciously compensated for abdominal muscle weakness by maintaining LM activation to empower spinal stability. However, this should be considered a hypothesis rather than a firm interpretation, as it is not directly supported by our data.

The strength of the association was measured using Spearman’s correlation coefficient (r_s_), which can be classified as follows: no correlation (r_s_ = 0), very weak or negligible (0 < r_s_ ≤ 0.19), weak (0.2 ≤ r_s_ ≤ 0.39), moderate (0.40 ≤ r_s_ ≤ 0.59), strong (0.6 ≤ r_s_ ≤ 0.79), very strong (0.8 ≤ r_s_ ≤ 1), and monotonic correlation (r_s_ = 1), regardless of the direction (positive or negative) [57]. This study identified non-significant associations between NSLBP intensity and core muscle strength in both groups. According to the previous classification of the association’s strength, in the CD group, LM strength showed a weak negative association with pain (r_s_ = −0.371), suggesting a potential trend where weaker LM may contribute to higher NSLBP, though this was not statistically significant. TrA strength exhibited a negligible positive association (r_s_ = 0.097), indicating no meaningful relationship with pain levels. In the VD group, TrA strength showed a weak positive association (r_s_ = 0.354), while LM strength showed a negligible negative association (r_s_ = −0.039) with pain levels. Association testing was reinforced by a multiple regression analysis, which confirmed the non-significant association and the poor model fit between NSLBP VAS scores and all other variables in our model. The results of this regression mean that this particular set of variables (age, BMI, parity, and core muscle strength) does not explain NSLBP levels well. The non-significant associations may partly reflect a risk of type II error due to the limited sample size, rather than a true absence of association. This can guide future research by focusing on the need to explore different predictors. We compared our findings with studies investigating the association between LBP and core muscle strength in musculoskeletal dysfunctions. Our results are in line with those of a systematic review by Roffey et al. [58], which concluded that high-quality evidence from six studies suggested non-significant associations between LBP and awkward postures, such as those related to weaknesses in the transverse abdominis (TrA) and lumbar multifidus (LM) muscles. Conversely, our results differ from those of Abdelraouf & Abdel-Aziem [59], who explored the relationship between core muscle endurance (a special type of muscle strength) and NSLBP in athletes. They reported a significant moderate association and concluded that poor core endurance is likely linked to NSLBP in athletes. The explanation for this disagreement may be related to the difference between the participants in the two studies. Athletes have higher physical demands, greater core muscle engagement and endurance, and potentially different neuromuscular adaptations compared to postpartum women, who are exposed to pronounced biomechanical consequences and hormonal changes specific to pregnancy and postpartum periods.

This study has several strengths. First, it is the first to assess the intensity of NSLBP and the strength of core muscles (TrA and LM) in women who experienced CD or VD at 6–12 weeks postpartum, while also testing the association between these variables. Second, the comparison between the CD and VD groups helped eliminate the confounding effect of pregnancy on the outcome variables. Third, a high level of standardization was maintained throughout the study, as the same researcher consistently collected the data. Fourth, valid instrumentation was used to assess the outcome variables. However, this study also has several limitations. First, the cross-sectional design could be a weakness due to the absence of pre- and post-exposure assessments for the outcome variables. Second, we used the VAS, which provides a self-reported measure of pain intensity rather than an objective biomarker, and future studies should complement VAS with additional measures such as pressure pain thresholds or functional disability indices (e.g., Oswestry Disability Index) to enhance robustness. Also, the PBU measures core muscle strength indirectly and should not be the sole assessment tool; it should be augmented by fine-wire electromyography and ultrasound imaging. Third, the evaluation of diastasis recti abdominis was not considered as an Inclusion or exclusion criterion, despite its potential association with abdominal muscle strength [60]. Fourth, we did not use imaging to rule out structural or degenerative causes of NSLBP, and our analysis was restricted to testing associations with TrA and LM muscle strength rather than implying causality.

Our findings suggest important clinical implications for postpartum rehabilitation. Women who underwent CD may experience greater TrA weakness and higher NSLBP intensity compared to those who had VD. These findings represent associations rather than causal relationships, but they highlight the potential value of incorporating TrA-focused rehabilitation strategies for this population. Also, the non-significant associations between NSLBP intensity and core muscle strength underscore the multifactorial nature of postpartum NSLBP, where altered movement patterns, biomechanical changes, and neuromuscular dysfunction may play a more critical role than isolated core muscle deficits. Therefore, rehabilitation should not focus solely on strengthening TrA or LM but should incorporate functional movement training, postural correction, and pain modulation modalities like TENS or Interferential current therapy. Individualized treatment plans should be based on delivery mode, with post-CD women requiring more targeted TrA re-education exercises.

We fully acknowledge that trunk stability involves a broader muscular network, including global abdominal muscles, erector spinae, diaphragm, and pelvic floor. Evaluating all of these muscles together would indeed provide a more comprehensive understanding. We aim to expand this line of research in future prospective studies, incorporating imaging and EMG techniques to assess a wider range of muscles and their synergistic role in postpartum spinal stability. Future research should adopt a prospective design to allow for pre- and post-exposure assessments, ensuring a more accurate measurement of the impact of delivery mode on core muscle strength and NSLBP and their relationship. Multicenter studies with larger sample sizes are necessary to enhance generalizability. Also, Future studies could examine the long-term effects of delivery mode on core muscle strength, posture, and NSLBP.

## 5. Conclusions

Women who underwent CD experienced a greater increase in NSLBP and a significant decrease in TrA strength compared to those who had VD, while LM strength remained unaffected by the mode of delivery. Non-significant associations were found between NSLBP and core muscle strength in either group. Based on these associations, TrA-specific training may be a valuable component of postpartum rehabilitation, particularly for women following CD. Electrophysical modalities such as TENS and interferential current therapy could also be considered as supportive options for managing postpartum NSLBP. Future prospective studies with larger sample sizes are needed to confirm these associations and refine postpartum rehabilitation strategies.

## Figures and Tables

**Table 1:** Comparison of the general characteristics between groups A and B.

Items	Physical Characteristics of Subjects (Mean ± SD)		
	Age in Years	BMI in kg/m^2^	Parity (Number of Deliveries)
Group A (*n* = 18 CD Women)	25.8 ± 5.6	24.9 ± 3.1	2.2 ± 1.2
Group B (*n* = 18 VD Women)	27.3 ± 3.5	24.2 ± 2.4	2.2 ± 1.3
*p*-value	0.342	0.429	0.894

Group A: CD women; Group B: VD women. Data are expressed as mean ± standard deviation (SD).

**Table 2:** The Unpaired *t*-test between A and B for the VAS scores.

VAS Scores of NSLBP (Mean ± SD)	
Group A (*n* = 18 CD Women)	5.1 ± 1.5 (95% CI: 4.4–5.8)
Group B (*n* = 18 VD Women)	3.4 ± 0.8 (95% CI: 3.0–3.8)
MD (change)	1.7
*p*-value	0.000 *

Group A: CD women; Group B: VD women. Data are expressed as mean ± standard deviation (SD). MD: mean difference; CI: confidence interval; *: significant, *n*: number.

**Table 3:** The Unpaired *t*-test between the 2 groups for TrA MVIC and LM MVIC.

TrA MVIC (Mean ± SD)		LM MVIC (Mean ± SD)
Group A (*n* = 18 CD Women)	2.8 ± 1.1 (95% CI: 2.2–3.4)	3.7 ± 0.6 (95% CI: 3.4–4.0)
Group B (*n* = 18 VD Women)	3.7 ± 0.5 (95% CI: 3.4–3.9)	3.7 ± 0.5 (95% CI: 3.5–4.0)
MD (change)	−0.9	0
*p*-value	0.009 *	0.602

Group A: CD women; Group B: VD women. Data are expressed as mean ± standard deviation (SD). MD: mean difference; CI: confidence interval; *: significant, *n*: number.

**Table 4:** Association between VAS and each core muscle strength for each group.

Correlations					
			VAS	TrA MVIC	LM MVIC
Spearman’s rho	Group A VAS	Correlation Coefficient	1.000	0.097	−0.371
		Sig. (2-tailed) *p*-value	-	0.702	0.129
Spearman’s rho	Group B VAS	Correlation Coefficient	1.000	0.354	−0.039
		Sig. (2-tailed) *p*-value	-	0.149	0.877

Group A: CD women; Group B: VD women.

**Table 5:** Multiple linear regression model.

Model	R	R Square	Adjusted R Square	F Change	df1	df2	Sig. F Change
All predictors (Age, BMI, Parity, TrA MVIC, LM MVIC)	0.443	0.196	0.062	1.464	5	30	0.231

**Table 6:** Multiple linear regression coefficients.

Model	Standardized Coefficients Beta	*p*-Value	95% Confidence Interval (CI) for Beta		Approximated Effect Size (Semi-Partial r^2^)
			Lower Bound	Upper Bound	
(Constant)		0.645	−4.717	7.498	
Age	−0.022	0.915	−0.137	0.124	Negligible
BMI	0.353	0.087	−0.029	0.397	Small to moderate
Parity	0.088	0.638	−0.334	0.537	Negligible
TrA MVIC	−0.221	0.205	−0.863	0.193	Small
LM MVIC	−0.062	0.715	−1.083	0.752	Negligible

## Data Availability

The data from this study can be obtained upon request from the corresponding author. Due to ethical considerations, the data are not publicly accessible.

## Abbreviations

The following abbreviations are used in this manuscript:

NSLBP	Nonspecific low back pain
VAS	Visual analogue scale
TrA	Transversus abdominis
LM	Lumbar multifidus
PBU	Pressure biofeedback unit
CD	Cesarean delivery
VD	Vaginal delivery
BMI	Body mass index
WHO	World Health Organization
CAPMAS	Central Agency for Public Mobilization and Statistics
